# Supplementary figures and images for: Relationship between Acropora millepora juvenile fluorescence and composition of newly established Symbiodinium assemblage
Source: PeerJ. 2018 Jun 15;6:e5022. doi: 10.7717/peerj.5022 (PMC6005160; doi:10.7717/peerj.5022)

A

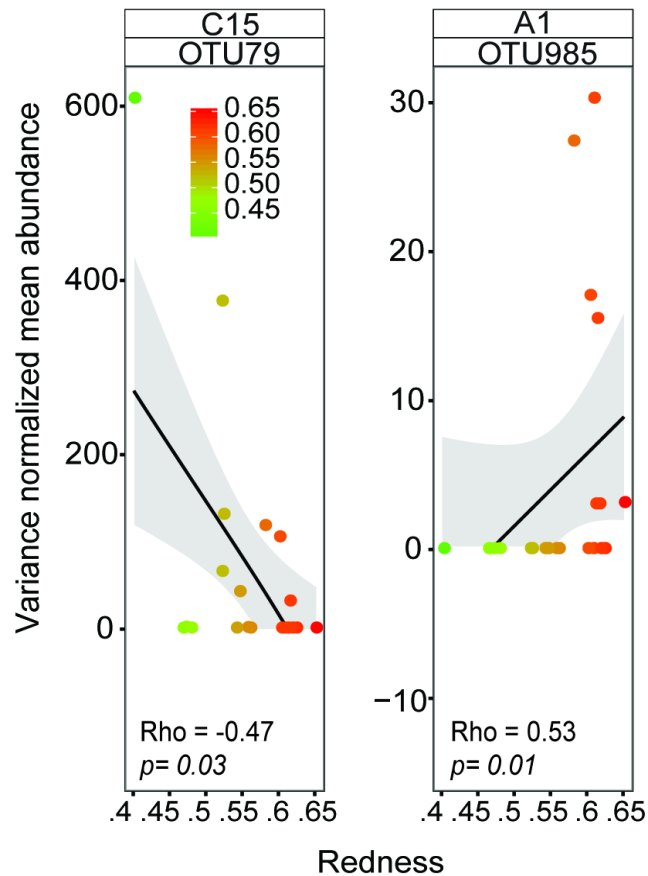

B

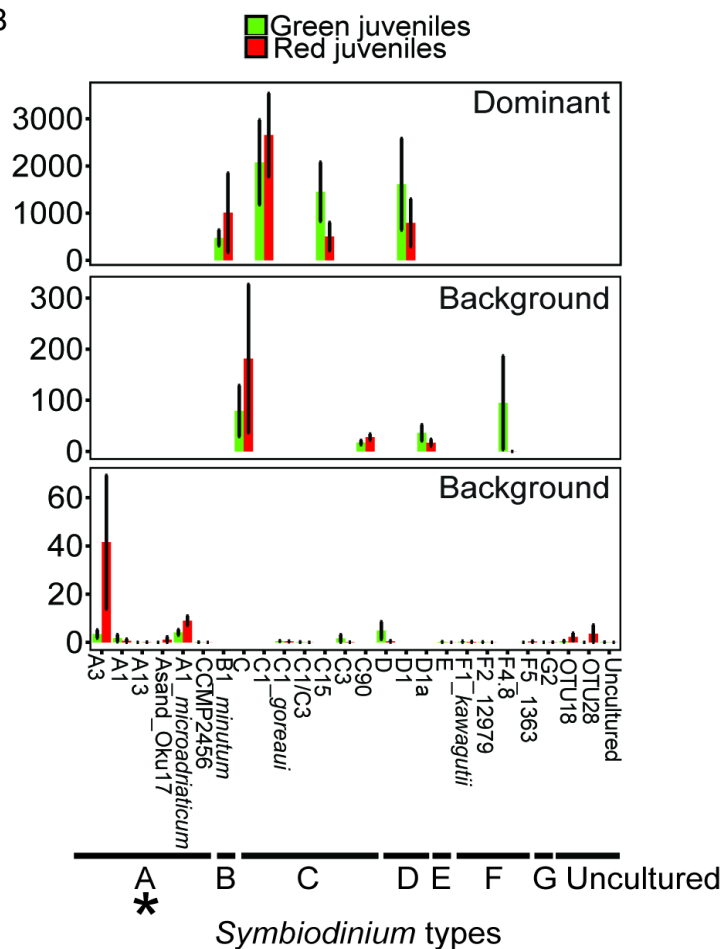

Supplement: Figure S1 — (A) Barplots of clades/type/OTUs. (B) Scatterplots comparing the redness of juveniles (x-axis) to the variance normalized abundance of different Symbiodinium types (y-axis, depicted as whole numbers). Asterisks below clade designations on barplots are significantly correlated with red juveniles. [file peerj-06-5022-s001.pdf]
