# Supplementary material for: Relationship between Acropora millepora juvenile fluorescence and composition of newly established Symbiodinium assemblage
Source: PeerJ. 2018 Jun 15;6:e5022. doi: 10.7717/peerj.5022 (PMC6005160; doi:10.7717/peerj.5022)
Supplement: Table S1 — Sample size per Symbiodinium clade/category per treatment group. * denote NCBI given names to OTU taxonomies, not author designations. [file peerj-06-5022-s002.docx]

**Table S1**. Sample size per *Symbiodinium* clade/category per treatment group. * denote NCBI

given names to OTU taxonomies, not author designations.

|  | **N° OTUs** | **N° red** | **N° green** |
| --- | --- | --- | --- |
| A | 19 | 228 | 190 |
| B | 2 | 24 | 20 |
| C | 28 | 336 | 280 |
| D | 10 | 120 | 100 |
| Ex-situ* | 11 | 132 | 110 |
| F | 2 | 24 | 20 |
| G | 1 | 12 | 10 |
| RCC2640*** | 6 | 72 | 60 |
| scyphozoan_medusae* | 1 | 12 | 10 |
| Uncultured*** | 9 | 108 | 90 |
