# Supplementary material for: Relationship between Acropora millepora juvenile fluorescence and composition of newly established Symbiodinium assemblage
Source: PeerJ. 2018 Jun 15;6:e5022. doi: 10.7717/peerj.5022 (PMC6005160; doi:10.7717/peerj.5022)
Supplement: Table S2 — * denote NCBI given names to OTU taxonomies, not author designations. [file peerj-06-5022-s003.docx]

**Table S2**. Sample size per *Symbiodinium* type per treatment group. * denote NCBI given

names to OTU taxonomies, not author designations.

|  | **OTUs** | **N° red** | **N° green** |
| --- | --- | --- | --- |
| A13 | 1 | 12 | 10 |
| A3 | 3 | 36 | 30 |
| Asand_Oku17*** | 1 | 12 | 10 |
| CCMP2456*** | 1 | 12 | 10 |
| *S. microadriaticum* | 13 | 156 | 130 |
| *S. minutum* | 2 | 24 | 20 |
| C | 7 | 84 | 70 |
| C1 | 8 | 96 | 80 |
| C1/C3_Two0501-8 | 1 | 12 | 10 |
| C15 | 8 | 96 | 80 |
| C3 | 3 | 36 | 30 |
| C90 | 1 | 12 | 10 |
| D | 3 | 36 | 30 |
| D1 | 2 | 24 | 20 |
| D1a | 5 | 60 | 50 |
| *Amphisorus.hemprichii** | 3 | 36 | 30 |
| *Montipora.digitata** | 1 | 12 | 10 |
| *Zoanthus.sociatus** | 7 | 84 | 70 |
| F2_12979 | 1 | 12 | 10 |
| F5_1363 | 1 | 12 | 10 |
| G2 | 1 | 12 | 10 |
| RCC2640*** | 6 | 72 | 60 |
| scyphozoan_medusae_1Sy24*** | 1 | 12 | 10 |
| OTU18 | 3 | 36 | 30 |
| OTU28 | 1 | 12 | 10 |
| Uncultured*** | 5 | 60 | 50 |
