# Supplementary material for: Relationship between Acropora millepora juvenile fluorescence and composition of newly established Symbiodinium assemblage
Source: PeerJ. 2018 Jun 15;6:e5022. doi: 10.7717/peerj.5022 (PMC6005160; doi:10.7717/peerj.5022)
Supplement: Supplemental Information 1 [file peerj-06-5022-s004.docx]

**Supplementary Methods**

“Relationship between *Acropora millepora* juvenile fluorescence and composition of newly established *Symbiodinium* assemblage”

KM Quigley, ME Strader & MV Matz

**Construction of custom Blast+ library**

We would refer the reader to NCBI’s Blast Command Line Applications User Manual (<https://www.ncbi.nlm.nih.gov/books/NBK279690/>) for full instruction as these steps may vary per user depending on the HPC cluster.

Download and extract full NCBI database:

1. Make sure blastn is in your path and check the version
2. Download the nt database from the NCBI ftp website (location and size may change).
3. Unpackage and unzip the files in that directory following the instructions here (<http://www.ncbi.nlm.nih.gov/books/NBK52640/>)
   - Inflating the compressed archive and extracting the tar file will regenerate the files for this database. To save disk space, the original .gz file can be removed after the installation.
4. Make sure nt.nal file is there.
   - The nt.nal is the file that ties all the database (db) together, make sure that is in your home database folder.
5. Check the contents of the unzipped database folder, should be 10 documents for each of the 26 zipped files (but this will change as NCBI grows so make sure to check latest online size!)
6. Database check time. This might show a couple of errors first depending on your level of permissions. Make sure you are in the directory were your database is when you are doing this.
   - blastdbcheck -db nt
7. Downloaded the taxonomy zipped file from here (<http://www.ncbi.nlm.nih.gov/books/NBK1763/>). Place it in the db folder (MUST BE IN SAME DIRECTORY AS THE database files!)
8. Unzip/untarr the taxonomy file
   - tar zxvpf taxdb.tar.gz
9. Database check time again, just to be sure

Make specific database from full NCBI database:

1. Download a list of organism-specific GI identifiers from entrez (<http://www.ncbi.nlm.nih.gov/nuccore/>)
2. Create new database of specific GI identifies for you organisms of interest.
   - blastdb_aliastool -db nt -gilist ~/org.gi.txt -dbtype \nucl -out nt_org -title "nt_org"
3. Makes sure it is the size you think it should be with correct number of nucleotide sequences- sometimes some are filtered due to redundancy (that is ok).
4. You can now search against this new database using the blastn call.
